# The Great Ormond Street Hospital immunoadsorption method for ABO-incompatible heart transplantation: a practical technique

**DOI:** 10.1177/0267659120926895

**Published:** 2020-06-03

**Authors:** Richard Issitt, Richard Crook, Michael Shaw, Alex Robertson

**Affiliations:** 1 Perfusion Department, Great Ormond Street Hospital, London, UK; 2 Institute of Cardiovascular Science, University College London, London, UK

**Keywords:** immunoadsorption, heart transplantation, isohaemagglutinins, cardiopulmonary bypass

## Abstract

Traditionally, ABO-incompatible heart transplantation was accomplished using a plasma exchange technique to remove recipient plasma containing donor-incompatible anti-A/B isohaemagglutinins. However, this technique exposed patients to large volumes of allogeneic blood and blood products (up to three times the patient’s circulating volume). In 2018, we published the first reported case of an ABO-incompatible heart transplant using an intraoperative immunoadsorption technique which minimises the exposure to blood products by specifically targeting anti-A/B isohaemagglutinins. We have subsequently used this technique in all children undergoing ABO-incompatible heart transplantation and become convinced of its efficacy in this population while observing no adverse effects. This article outlines the practical details required to perform the technique in order to avoid hyperacute rejection.

## Introduction

The concept and first practical method of ABO-incompatible (ABO-i) heart transplantation was introduced by West et al.^1^ in 2001. Using a plasma exchange technique, undertaken before the onset of cardiopulmonary bypass (CPB) (which utilises blood cells matched to the recipient, and plasma and platelets matched to the donor), the immaturity of the recipient’s immune system was exploited to enable the crossing of the ABO blood group barrier.^2,3^ This technique, however, entailed a period of potential haemodynamic instability where mechanical support could not be initiated without sacrificing the efficacy of the plasma exchange process.^3^ Initial work suggested that the technique was limited to the first 12-14 months of life as after this isohaemagglutinin titres were higher and risked acute rejection. Further work by the West group and others saw this time frame extended well beyond this point, with the oldest reported successful procedure carried out in a 5-year-old girl in Sweden.^3–5^ While highly successful, showing comparable outcome data to ABO-compatible heart transplantation,^6^ the procedure exposed patients to vast quantities of allogeneic blood and blood products (at least three times the patient’s circulating volume), increasing the risk of transfusion-related morbidity.^3,7^ This is especially prevalent in patients >10 kg where the required volumes for plasma exchange can exceed 3 L, thereby substantially increasing the number of donor exposures. To address this, we sought to target the anti-A/B isohaemagglutinins directly, through the process of immunoadsorption (IA), rather than indirectly as a consequence of displacing the patient’s blood volume. We reasoned this would have a number of benefits. First, the process could be undertaken during the operation, as opposed to extending the period in theatre which is necessary for the plasma exchange process, thus avoiding the period of potential instability. Second, by avoiding the massive transfusion requirements, disruption to homeostasis is avoided, and the physiological insult and subsequent morbidity are minimised. This approach resulted in accomplishing ABO-i heart transplants with a substantial reduction in blood and blood product transfusion while also avoiding hyperacute rejection.^8^

The purpose of this communication is to describe the technique in more practical detail for the benefit of other centres wishing to utilise this process.

## Methods

### Standard setup

**Figure 1.**
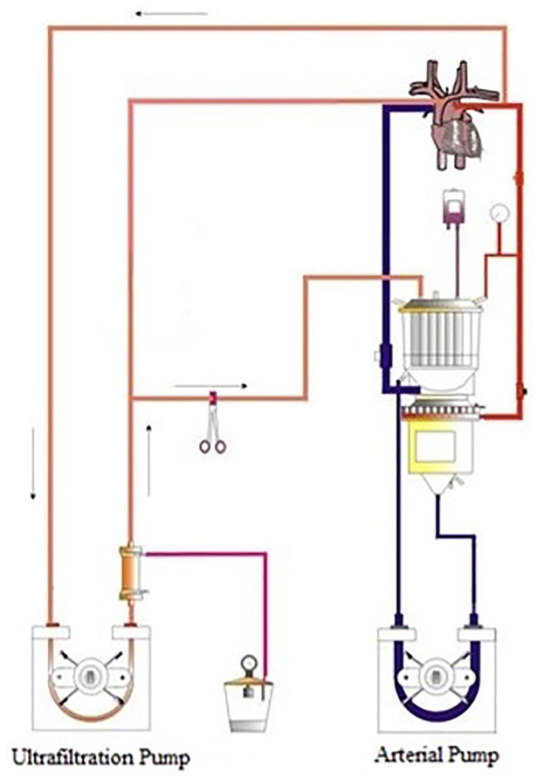
Standard CPB setup. Blood for filtration is taken via the ultrafiltration pump from the arterial limb of the bypass circuit. The filtrate is removed under vacuum and the haemoconcentrated blood is returned to systemic circulation via the venous reservoir or back to the right atrium via a wye (Y) connector for performing modified ultrafiltration.

Our standard CPB setup is using a S5 heart-lung bypass machine (Stockert; LivaNova, Munich, Germany), with a mast-mounted single arterial pump (150 mm diameter) and two mast-mounted double-headed pumps (85 mm diameter). These are used for extra-cardiac suction and intra-cardiac venting and haemofiltration. There is also a base-mounted double-headed pump (85 mm diameter) for cardioplegia delivery. As our haemofiltration line arises from the arterial limb of the CPB circuit (Figure 1), the haemofiltration pump is slaved to the main arterial pump to prevent cavitation during modified ultrafiltration. For a typical patient of 5 kg undergoing ABO-i heart transplantation, the CPB circuit consists of a tubing set with a 3/16″ arterial line and 1/4″ venous line (LivaNova) with an oxygenator with hardshell venous reservoir (CAPIOX^®^ FX05; Terumo, Leuven, Belgium). The haemofiltration circuit described above takes blood from the arterial line and returns it, via a wye (Y) connector, either to the venous reservoir or, via a 1/8″ line, to the right atrium for modified ultrafiltration as previously described.^9^

### ABO-i IA modified circuit

**Figure 2.**
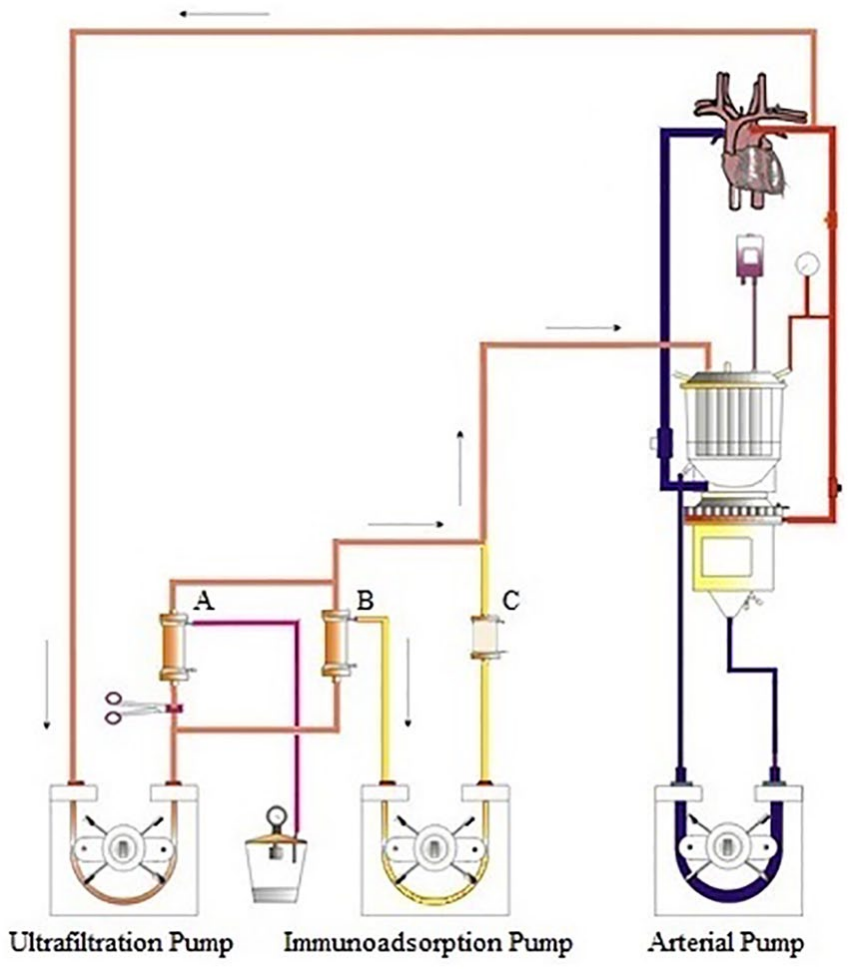
CPB circuit with integrated immunoadsorption column. Whole blood is pumped, using the ultrafiltration pump, from the arterial limb of the bypass circuit via the plasma separator (B). The haemofilter (A) is clamped from the circuit at this stage, having been used for pre-bypass ultrafiltration and later for conventional and modified ultrafiltration. The separated plasma is then pumped through the anti-A/B immunoadsorption column (C) via the immunoadsorption pump. The haemic content from the plasma separator outlet is reconstituted with the anti-A/B depleted plasma and returned to the circulation via the venous reservoir.

To facilitate anti-A/B isohaemagglutinin removal, plasma must be separated from the circulating volume. To the above circuit, a plasma separator (Asahi Kasei PS-03; LINC Medical Systems Ltd, Leicester, UK) is placed in parallel to the haemofilter (HF-06; LivaNova) using a positive screw locking (POS lock)–ended wye connector to the haemofiltration line (Figure 2). Distal to both the plasma separator and haemofilter, a second POS lock–ended wye connector is used to recombine the two streams. This, in turn, is then further split via a wye for return to the venous reservoir or via a 1/8″ line to the right atrium as described above.

The rate of effluent (plasma) flow must be controlled, and so an additional roller pump (described here as the IA pump; 150 mm diameter) is attached to the mast with the haemofiltration pump. This takes the separated plasma to the anti-A/B IA column (Glycosorb^®^-ABO Anti-A/B Specific Column; Glycorex Transplantation AB, Lund, Sweden), which removes both anti-A and anti-B isohaemagglutinins. The post–plasma separator blood and post–IA column plasma are then reconstituted into a single line returning to the systemic circulation via the venous reservoir. In order to prevent a mismatch in flow through the plasma separator, and effluent plasma flow to the IA column, the IA pump is slaved to the haemofiltration pump and locked to prevent a faster flow rate in the former.

### Priming

Following priming of the main systemic CPB circuit, flow is then initiated through the haemofilter, and once de-aired is clamped off from the circuit. Flow is then passed through the plasma separator at an initial rate of 50 mL/min and increased to 200 mL/min over 5 min. This is the maximum recommended rate for the plasma separator described here. Once the plasma separator is de-aired, the IA pump is started and increased to a maximum flow rate of 40 mL/min over 5 min. This is the maximum recommended rate for the anti-A/B IA column described here. Once fully primed, both circuits can be clamped off ready for initiation of CPB. The manufacturer stipulates that air must not be allowed to enter the IA column during circuit priming. Therefore, the inflow to the column can either be detached as the line fills with fluid from the plasma separator or a three-way stopcock may be placed at the inflow to the column to remove air from the line until it is filled with fluid.

### CPB management

Once CPB has been established, the flows through the plasma separator and subsequently the IA column can be increased to the rates outlined above. This can be left to run for the required duration of treatment before reperfusion of the donor organ occurs (discussed in the following section) and for the remainder of the CPB run following X-clamp removal (though the target titre of 1:2 or better should be achieved prior to reperfusion). Due to the shunting of arterial flow required for this technique, the flow rate of the main arterial pump should be increased to compensate. During the setup of the circuit, it is vital to account for this when calculating cardiac index as this will influence the selection of arterial pump boot size necessary.

A word of caution should also be noted should haemofiltration be required during the IA process, especially if vacuum is applied to the haemofiltration effluent line; this can cause preferential blood flow through the haemofilter at the expense of the plasma separator. This results in a reduction of separated plasma for the IA pump, despite a constant flow being maintained on the effluent of the plasma separator. To reduce the possibility of this occurring, a flow probe should be placed on the blood outlet of the plasma separator and the haemofiltration pump flow increased to compensate to ensure sufficient blood flow through it and thus prevent rupturing of the plasma separator fibres that may occur otherwise.

### Duration of IA treatment

We have previously covered the issue of patient suitability and selection, so will not discuss here.^8^ The recommended treatment time (the length of IA prior to reperfusion of the donor organ) is calculated as follows

$$\begin{array}{l} \mathrm{Minimum}\;\mathrm{treatment}\;\mathrm{time}\;(\min)=\\ \;\;\;\;\frac{\left(PaV(\mathrm{mL})+\;TPV(\mathrm{mL})\right)*\left(\frac{\left(100-Hct\right)}{100}\right)}{40\;\mathrm{mL}/\min}*nPV \end{array}$$

where *PaV* is the patient’s circulating volume, *TPV* is the total prime volume, *Hct* is the patient’s haematocrit and *nPV* is the number of plasma volumes for treatment

Based on our previous experience, the recommended number of PV that passes through the IA column should equal the number of titre reductions required to ensure a maximum final concentration of 1:2. For instance, a patient with a starting titre of 1:32 requires a minimum of four PV passes through the IA column before reperfusion of the donor organ occurs. Therefore, the calculated rate multiplied by the number of PV passes required equals the minimum duration of IA treatment. An example is a 5-kg child with a circulating volume of 425 mL, 30% haematocrit and titre of 1:32 undergoing this process with a 450-mL total prime volume

$$\mathrm{Minimum}\;\mathrm{treatment}\;\mathrm{time}=\frac{425+450*\left(\frac{\left(100-30\right)}{100}\right)}{40\;\mathrm{mL}/\min}*4$$

$$\mathrm{Minimum}\;\mathrm{treatment}\;\mathrm{time}=\frac{875\;\mathrm{mL}*0.7}{40\;\mathrm{mL}/\min}*4$$

Minimumtreatmenttime=62min

While in practice, due to the dilutional effects of the CPB volume, the isohaemagglutinin titre drops following initiation of CPB, we do not adjust the calculations to reflect this, preferring instead to accept the overestimation of the process.

## Conclusion

The process of ABO-i heart transplantation has been utilised for over 20 years with excellent outcomes comparable to ABO-compatible transplantation.^10^ However, the initially described methodology of plasma exchange transfusion puts patients at increased risk of transfusion-related morbidity due to the significant volumes of blood and blood products required.^7^ The modification to the methodology described here has the potential to drastically reduce this impact. In the first reported case using this technique, the patient received two units (520 mL) of packed red blood cells and one unit (200 mL) of plasma. Had they undergone the plasma exchange method, the patient would have received eight units of packed red cells (~2,000 mL) and 10 units (2,000 mL) of plasma in a 1:1 ratio, significantly increasing donor exposure and subsequent risk of transfusion-related morbidity.^8^

We believe that this technique has several positive implications for paediatric heart transplantation. First, we have previously shown that the process is predictable and efficient, allowing for planning of time needed for isohaemagglutinin removal before donor organ reperfusion.^8^ Second, patients have less blood product exposure and are not subject to the hemodynamic instability from fluid shifts associated with the plasma exchange technique.^3,8^ Finally, and perhaps most significantly, we believe that this technique has the potential to expand the application of ABO-i heart transplantation to larger children and those recipients with higher anti-A/B isohaemagglutinin titres than traditionally treated, although more research is needed to explore this. Given the increasing demand for donor hearts within a relatively constant or even falling level of donor organ availability, we hope this method may assist in maximising the effective donor pool for our patients.^11^
